# Does COVID-19 persistently affect educational inequality after school reopening? evidence from Internet search data in China

**DOI:** 10.1371/journal.pone.0293168

**Published:** 2023-10-30

**Authors:** Xuejing Hao

**Affiliations:** School of Economics and Management, University of Science and Technology Beijing, Beijing, China; Guangzhou Institute of Geography, Guangdong Academy of Sciences, CHINA

## Abstract

The literature has extensively documented how Covid-19 affects educational inequality, but it remains unclear whether such an effect persists after school reopening. This paper attempts to explore this issue by investigating the search gap for learning resources in China. I categorized learning resources into four types: “school-centered resources”, “parent-centered resources”, “online tutoring agencies resources” and “in-person tutoring agencies resources”. Using Internet search data, I found that nationwide search intensity for learning resources surged when schools were closed, and such search behaviors remained after schools reopened. I also found that high socioeconomic status households had better access to school- and parent-centered resources, and online tutoring resources, even after schools reopened. Given its persistent impact on learning, the pandemic will likely widen educational inequality over extended periods.

## 1 Introduction

The global outbreak of the Covid-19 pandemic has led to school lockdowns, affecting nearly 1.6 billion school children worldwide at its peak [1]. School interruption may decrease students’ academic performance, especially for students from low socioeconomic status (Low-SES) households [2–5]. Unequal access to learning resources may be one of the main channels to widen such educational inequality [6,7]. As schools have gradually returned to normal, educators and policymakers are racing to get students back on track. For example, the U.S. government has allocated $122 billion in relief funds to support academic recovery [8]. To help such remedial interventions better target students in need in the post-Covid period, it is crucial to understand the persistent effects of Covid-19 on students’ unequal access to learning resources after school reopening.

China’s unique features offer a well-suited setting to explore this issue due to its unique following features. First, China took strict measures to control the spread of the pandemic in 2020 and 2021, and students largely resumed in-person schooling after late May 2020. This provides a larger time window to explore the persistent impact of the pandemic on learning. By contrast, schools returned to normal only in 2022 in many other countries. Second, China has the largest education system in the world, with a compulsory education system covering 158 million students in 2022. However, there are large regional education inequalities in China, and students in underdeveloped regions have limited access to learning resources [9,10]. This provides a unique setting for understanding the pandemic-induced educational inequality.

This paper used weekly Baidu Index (similar to Google Trends) data of China’s 361 prefecture-level cities to explore this issue. I used the difference-in-differences approach and event-study analysis to study the inequality in access to different learning resources during school closures, as well as whether the possible inequality persists after school reopening. I found that students living in high socioeconomic status areas (High-SES households) had an advantage over those living in low SES areas (Low-SES households) in access to school- and parent-centered resources, as well as online tutoring resources, even after schools reopened. By contrast, the inequality in access to in-person tutoring agencies resources decreased during school closures, but increased after schools reopened.

My main contributions are as follows. First, this study is among the first to examine whether Covid-19 has a lasting impact on educational inequality after school reopening [8,11,12]. While the literature [7,13–16] has extensively studied the impact of the pandemic on educational inequality, little has been known about whether such an effect persists after school reopening. China is a well-suited setting to explore the persistent impact of the pandemic on educational inequality because China resumed in-person schooling only several months after the outbreak in 2020. By contrast, many other countries closed schools for more than three semesters. While my estimates may be a lower bound due to the short period of school closures in China, my findings can help understand possible long-term educational consequences of the Covid-19 pandemic in other settings.

The closest to my study is Bacher-Hicks et al. [7]. This paper differs in the following two ways. First, this paper focused on the school reopening period to explore the persistent effect of COVID-19, while Bacher-Hicks et al. focused on the short-term effect during the school lockdown period and found increased inequality in access to online learning resources. Second, this paper provided a broader pattern of how the pandemic affects educational inequality by exploring inequality in access to other resources such as tutoring resources, while Bacher-Hicks et al. only divided learning resources into “school- and parent-centered resources”. It is important to understand the role of tutoring in alleviating or exacerbating educational inequality, especially for countries with prevailing tutoring cultures like China [17–21].

Second, this paper contributes to the literature on the impact of external shocks on child education. The literature has documented how pandemic-related or natural disaster-related school closures affect students’ learning. For example, the 1918 flu pandemic, the 2003 Severe Acute Respiratory Syndrome (SARS) epidemic, or earthquakes [22–26]. But we have not witnessed educational disruption on such a large scale, affecting 1.6 billion students in more than 190 countries for at least several months. This study contributes to the literature by studying the possible lasting consequences of such an external shock (the Covid-19 pandemic) on educational inequality.

The remainder of this paper proceeds as follows. Section 2 provides a literature review on pandemic-induced educational inequality. Section 3 provides information on the development of Covid-19 in China, how students access learning resources during the pandemic, and the tutoring culture in China. Section 4 describes data, which come from the Baidu Index (similar to Google Trend), and empirical strategy. Section 5 presents results on unequal access to learning resources. Section 6 discusses. Section 7 concludes.

## 2 Literature review

Students from Low-SES households experienced more learning loss than others due to Covid-19. A wide range of studies examined the association between school closure and increased education inequality [13–16,27–29]. For example, Engzell, Frey and Verhagen were among the first to examine the impact of Covid-19 school closures on academic achievement [13]. Using a natural experiment in the Netherlands, they found that an eight-week school closure could translate into the loss of as much as one-fifth of the school year. This loss was most pronounced among students from disadvantaged homes. Agostinelli and his colleagues [14] examined the effects of pandemic school closures on children’s education in the USA and found that high school students from low-income communities suffered 0.4 standard deviation learning losses after a one-year school closure. By contrast, children from high-income communities initially remained unscathed.

Only a few studies, however, had examined whether this equality persisted after school reopening [8,11,12]. Singh, Romero and Muralidharanuse explored the learning loss after school closures and the pace of recovery after schools reopened using primary children data in rural Tamil Nadu [11]. They found that students tested in December 2021 (18 months after school closure) showed significant learning deficits in math and language compared to their peers in the same villages in 2019. Two-thirds of this deficit was made up within 6 months after school reopening. Using a triple-differences strategy, Lichand and Alberto Doria estimated the causal medium-run impacts of keeping schools closed for longer during the pandemic in Brazil [12]. They contrasted changes in educational outcomes across municipalities and grades that resumed in-person classes earlier in Q4/2020 or only in 2021 and found that relative learning losses from longer exposure to remote learning did not fade out over time. In addition, studies demonstrated the long-term effects of external shocks on learning. For example, Andrabi, Daniels and Dasfour found that 4 years after an earthquake in Pakistan, household and adult outcomes had recovered but learning losses endured. Their learning losses were equivalent to 1.5 fewer years of schooling [30].

To sum up, previous studies have almost focused on what happens during school closures and found increased education inequality due to the pandemic. Several recent studies have indicated the possible long-term educational effects of the pandemic. Based on this, this study hypothesizes that school closures will increase education inequality in access to learning resources and this inequality will persist after school reopening.

## 3 Background

The pandemic outbreak in China led to school lockdowns. Online classes were implemented immediately, resulting in a substantial increase in demand for learning resources. This section first provides background information on school closures and reopening in China. It then describes various resources available during the pandemic, with a particular focus on tutoring agencies resources. Finally, this section provides an overview of the tutoring culture in China, shedding light on its potential impact on increased educational inequality during the pandemic.

### 3.1. The Covid-19 pandemic in China

The Covid-19 pandemic broke out in Wuhan, the capital city of Hubei province, in December 2019 [31]. Wuhan was locked down on January 23, 2020. Mobility restrictions were subsequently imposed on 14 other cities in Hubei and throughout China [32]. By the end of April 2020, China had preliminarily contained the spread of Covid-19 on the mainland. Since then, the Chinese government has adopted a zero-COVID policy to contain the pandemic until December 2022, when China roll back lockdowns and gradually fully reopen [33].

**Fig 1:**
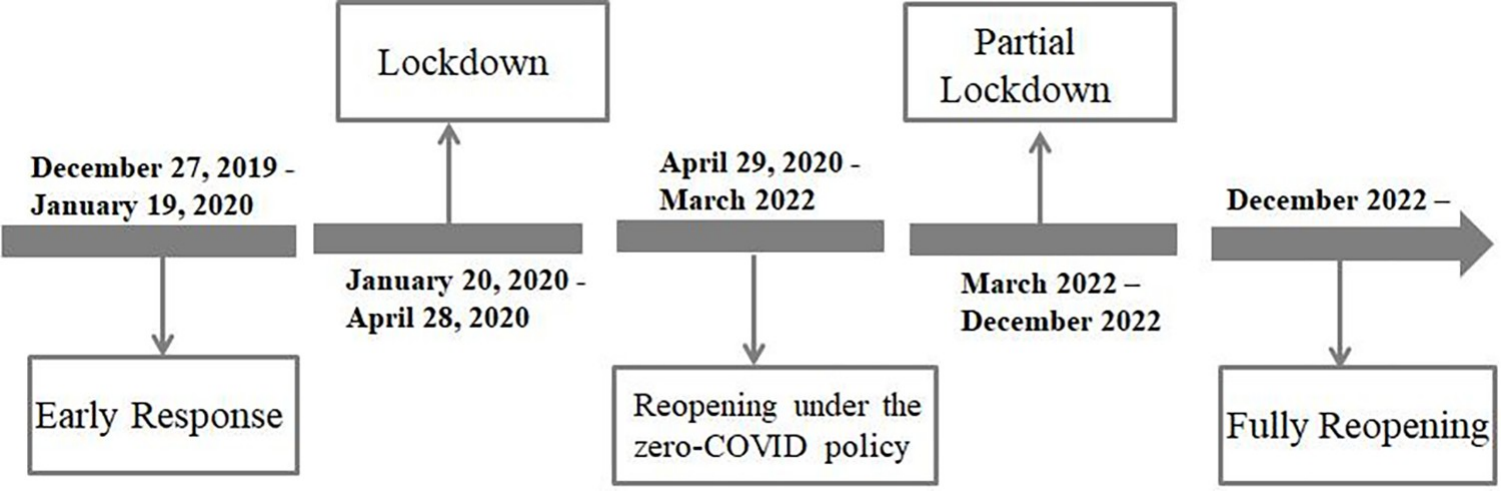
The five stages of China’s fight against the pandemic. Notes: This chart shows the four stages of China’s against the pandemic since December 2019. Timelines for the first two stages of the pandemic are from the white paper: *Fighting Covid-19*: *China in Action*. http://www.scio.gov.cn/zfbps/32832/Document/1681801/1681801.htm.

As Fig 1 shows, the pandemic in China has primarily gone through the following five stages (this study focus on the first three stages of the pandemic):

(1) The first stage is the early response period (December 27, 2019—January 19, 2020). During this period, cases of unexplained pneumonia were identified in Wuhan City, Hubei Province. China reported the pandemic and conducted epidemiological investigations to halt its spread. Although some studies suggest that the pandemic in Wuhan could start as early as early December 2019 [34], the pandemic timelines I show are intended to provide context for school closures and reopenings in China and should be interpreted with caution.

(2) The second stage is the lockdown period (January 20, 2020—April 28, 2020). The Chinese government adopted a lockdown strategy to contain the virus. Schools were closed during this period. The timing of school closures varies slightly by region, but is primarily concentrated in mid-February 2020.

(3) The third stage is the reopening period under the zero-COVID policy (April 29, 2020—March 2022). The pandemic situation was stable during this period, and the government conducted regular pandemic prevention and controls. Schools in most regions fully reopened by mid-May 2020.

(4) The fourth stage is the partial lockdown period (March 2022—December 2022). The outbreak of Omicron caused a Covid case surge in some regions, where partial lockdowns were implemented [35,36].

(5) The fifth stage is the fully reopening period (since December 2022). On December 7, the Chinese government adjust its prevention and control strategy and roll back lockdowns.

### 3.2. Learning resources during the pandemic

All Chinese schools were closed in February 2020 due to the Covid-19 outbreak and K-12 students turned to online learning to start their new spring semester. Governments and schools organized online classes promptly to mitigate possible negative consequences due to school closures. Online classes in most cities began in mid-February 2020 (see Table A1 in S1 File), the scheduled school start date, and continued until the end of May. After May 2020, almost all K-12 students resumed normal in-person classes. The average daily attendance of students and faculty was comparable to pre-pandemic levels.

Learning resources fall into four types during school lockdowns:

(1) “School-centered resources.” They represent education platforms that are necessary for students to take online courses during the pandemic [37]. These resources use video, communication, cloud storage, and other technologies to enable teachers to conduct online real-time teaching, upload resources, assign homework and interact with students, such as “Tencent Classroom” or “DingTalk”. DingTalk is an intelligent working platform created by Alibaba Group. Although originally designed for business purposes, it has been widely used by primary and secondary schools during school lockdowns in China.

(2) “Parent-centered resources.” They represent generic search terms indicating parents or students are seeking supplemental learning resources (such as “online learning” or “online education”). Parents or students are eager to make up for the school closures losses by searching online learning-related terms.

(3) “Online tutoring agencies resources.” They represent tutoring resources provided by agencies that focus on online learning, such as “Xueersi Online School” or “Yuanfudao”, which are Chinese top online tutoring agencies that provide a wide range of online tutoring services for K-12 students, covering subjects such as mathematics, English, and Science. In particular, many online tutoring organizations provided free online courses across the country during school lockdowns.

(4) “In-person tutoring agencies resources.” They represent tutoring resources provided by agencies that focus on offline learning, such as “Xueda Education” or “Jingrui Education”, which are Chinese top in-person tutoring agencies that offer in-person tutoring services for K-12 students. The government suspended offline business to slow the spread of the pandemic. Though some of them switched to online teaching temporarily, their businesses have been hit hard by the pandemic.

### 3.3. Tutoring culture in China

Tutoring culture prevails in East Asian societies like China, which are highly competitive and deeply rooted in the Confucian culture that promotes socioeconomic success through education attainment [17–21]. Many parents perceive supplementary tutoring as an effective way to improve students’ academic performance. For example, Zhang shows that private tutoring has a positive effect on urban students with lower achievement based on a survey of 6,043 12th graders in China [38].

The 2022 Global Education Monitoring Report released by UNESCO shows that more than 75% of Chinese primary and secondary school students participate in after-school tutoring [39]. The market size of Chinese K-12 tutoring is estimated to exceed 80 billion RMB (about 11.6 billion U.S. dollars) in 2017, involving more than 137 million students and 7 million teachers (http://www.cse.edu.cn/). However, there is a large inequality in access to tutoring resources in China, with students from high socioeconomic status (SES) households having an advantage over those from Low-SES households [40].

To shed light on how tutoring resources are used across households, I used survey data from the Present Situation of Extracurricular Training for K12 Students to show the tutoring use in China. This survey was conducted in 2017, with a large sample including 41,232 rural and 14,217 urban students [41]. As shown by Fig A1 in S1 File, I found that urban students were 17.64% more likely to participate in extracurricular tutoring than their rural counterparts. While the survey provided no direct information on in-person and online tutoring, I found that rural students were slightly more likely than urban students to prefer in-person tutoring. For example, 42% of students in rural areas preferred in-person tutoring, 4% higher than students in urban areas.

## 4 Methodology

### 4.1 Data

Data used in this study are free and publicly available online and do not involve human subjects; it is therefore not subject to institutional review board review requirements.

The main data were scraped from Baidu Index (https://index.baidu.com) using the Python. All search data were scraped based on a weekly basis and city level for the study period. Baidu Index is a data sharing platform based on Baidu’s (the largest Chinese search engine, similar to Google) massive netizen behavior data. I used the Baidu Index to query the weekly search intensity of keywords in 361 Chinese prefecture-level cities from January 2011 to January 2021. According to the official explanation, the Baidu Index value is not the actual search volume of a keyword, but a weighted sum of the number of searches. The weighting for calculating the Baidu index is a trade secret, so I don’t know what it is. However, the search index is likely to be linearly correlated with the number of searches for a keyword, as suggested by Qin and Zhu [42]. Therefore, the Baidu Index value may represent people’s online behavior without significantly distorting.

I used weekly search data of 40 keywords from January 2011 to January 2021. Table A2 in S1 File shows the list of these keywords (I first obtained 67 potential keywords and then selected the most popular 10 keywords respectively based on their rankings in nationwide search intensity [7], please see Tables A3-A6 in S1 File for detail). I divided them into four categories: (1) “school-centered resources”, which contains ten keywords indicating the top ten online education platforms (such as “Tencent Classroom” or “DingTalk”). (2) “Parent-centered resources”, which contains ten keywords indicating the top ten online learning-related general search terms (such as “online learning” or “online classes”). (3) “Online tutoring agencies resources”, which contains ten keywords indicating the top ten online tutoring agencies (such as “Yuanfudao” or “Xueersi Online School”). (4) “In-person tutoring agencies resources”, which contains ten keywords indicating the top ten in-person tutoring agencies (such as “Jingrui Education” or “Xueda Education”).

I added up the Baidu Index value of all relevant keywords under each learning resource to get the search intensity at the nation or city level. I further divided the search intensity value by the residential population to generate *SearchIntensity* at the city level [43]:

$$SearchIntensity_{it}=\frac{\;\sum_{j=1}^{10}Baidu\;searches\;for{\left[keyword\;j\right]}_{it}}{Residential\;population_{i}}$$ (1)

where *SearchIntensity*_*it*_ is the search intensity for a given city *i* in a given week *t*.

I used GDP per capita to measure city-level socioeconomic status (SES). City-level GDP and fixed broadband Internet access data come from the 2019 China Urban Statistics Yearbook (https://data.stats.gov.cn/easyquery.htm?cn=E0105), while the population data come from the Chinese 2010 census data (http://www.stats.gov.cn/sj/pcsj/rkpc/6rp/indexch.htm). Cities with GDP per capita above the median were classified as high SES regions. Table 1 compares the search intensity of all learning resources between high and low SES areas of the country.

**Table 1:** Mean weekly nationwide search intensity for learning resources.

Keyword Category			Pre-Covid	School Closures	School Reopening
**Overall Learning Resources**			51552	367299	196215
School-Centered Resources			27477	286516	135314
Parent-Centered Resources			2383	9954	4310
Online Tutoring Agencies Resources			14574	66086	50771
In-person Tutoring Agencies Resources			7118	4742	5819

Note: The table above contains the mean weekly nationwide search intensity for the four categories of keywords in the pre-Covid (before January 19, 2020), school closures (between January 20, 2020 and May 17, 2020), and school reopening (after May 18, 2020) periods. The “overall learning resources” represent the sum of the four categories of learning resources.

As the first row of Table 1 shows, after school closures due to the pandemic outbreak, the overall search intensity for learning resources increased dramatically. When schools reopened, the overall search intensity for learning resources decreased but still more than tripled compared to the pre-Covid level.

As rows (2)-(4) indicate, search intensity for “school-centered resources”, “parent-centered resources”, and “online tutoring agencies resources” increased significantly during the school closures period. When schools reopened, search intensity for these learning resources decreased but remained much higher than the pre-Covid level. By contrast, as shown in the final row, search intensity for “in-person tutoring agencies resources” decreased during school closures. When schools reopened, search intensity increased slightly but remained lower than the pre-Covid level.

### 4.2 Empirical model

I first used an event study specification to estimate the inequality in access to learning resources due to the Covid-19 pandemic. An event study is a statistical method used to examine the impact of a particular event on an outcome of interest over a defined event window. This method estimates the event effect by comparing the outcome when the event is in place to the outcome when it’s not [44]. It has been widely used in business and economics research [45]. In this study, an event study approach enables me to observe the dynamic effects of school closures and reopening on unequal access to learning resources over a given time period.

I interacted period dummies *PreCovid*_*Tt*_, *SchoolClosure*_*Tt*_, and *SchoolReopen*_*Tt*_ with *HighSES*, the dummy for whether a city is above median GDP per capita. My specification is as follows:

$$\begin{array}{l} SearchIntensity_{it}=\sum_{T=‐20}^{‐1}(\beta_{T}PreCovid_{Tt}+\eta_{T}PreCovid_{Tt}*HighSES_{i})\\ +\sum_{T=1}^{17}(\beta_{T}SchoolClosure_{Tt}+\eta_{T}SchoolClosure_{Tt}*HighSES_{i})\\ +\sum_{T=18}^{52}(\beta_{T}SchoolReopen_{Tt}+\eta_{T}SchoolReopen_{Tt}*HighSES_{i})\\ +\alpha_{1}PriorYears_{t}+\alpha_{2}PriorYears_{t}*HighSES_{i}+\mu_{w(t)}+\lambda_{y(t)}+\varepsilon_{it} \end{array}$$ (2)

where *SearchIntensity*_*it*_ is the search intensity for a given city *i* in a given week *t*. The week that the Wuhan lockdown began (starting with January 20, 2020) is represented by week zero (*t* = 0). *PreCovid*_*Tt*_, *SchoolClosure*_*Tt*_, and *SchoolReopen*_*Tt*_ represent the pre-Covid period (before January 19, 2020), the school closures period (between January 20, 2020 and May 17, 2020), and the school reopening period (after May 18, 2020), respectively.

Weekly indicators covers 72 weeks except January 20, 2020, including pre-Covid 2019 fall semester and post-Covid 2020 spring and fall semesters. My main coefficient of interest is *η*_*T*_, the difference in weekly search intensity between High- and Low-SES households during school closures and reopening periods. *PriorYears*_*t*_ represents the prior eight years of data, which is used to identify the week of year effects. *μ*_w(t)_ (i.e., 1–52) are week fixed effects to control for potential seasonal trends, *λ*_y(t)_ (i.e., 2011–2021) are year fixed effects to remove variation from year trends. I used clustered standard errors at the city level.

I next replaced the weekly *PreCovid*_*Tt*_, *SchoolClosure*_*Tt*_, and *SchoolReopen*_*Tt*_ in Eq (2) with *SchoolClosure*_*t*_ and *SchoolReopen*_*t*_ to make the specification become a difference-in-difference (DID) form. A difference-in-differences method allows this study to estimate the causal effect of school closures and reopening on education inequality by measuring changes in search intensity for High- and Low-SES households. The specification is as follows:

$$\begin{array}{l} SearchIntensity_{it}=\beta_{1}SchoolClosure_{t}*HighSES_{i}+\beta_{2}SchoolClosure_{t}*LowSES_{i}\\ +\beta_{3}SchoolReopen_{t}*HighSES_{i}+\beta_{4}SchoolReopen_{t}*LowSES_{i}\\ +\alpha HighSES_{i}+\mu_{w(t)}+\lambda y(t)+\varepsilon_{it} \end{array}$$ (3)

where *SchoolClosure*_*t*_ and *SchoolReopen*_*t*_ represent the school closures period (between January 20, 2020 and May 17, 2020), and the school reopening period (after May 18, 2020), respectively. *β*_1_ and *β*_2_ represent the change in search intensity for learning resources for High- and Low-SES households during school closures, respectively; *β*_3_ and *β*_4_ represent the change in search intensity for learning resources for High- and Low-SES households after school reopening, respectively.

## 5 Results

In this section, I will present the results about the changes in the nationwide search intensity for learning resources and about the inequality in access to learning resources between households living in high and low SES areas.

### 5.1 Nationwide search intensity

**Fig 2:**
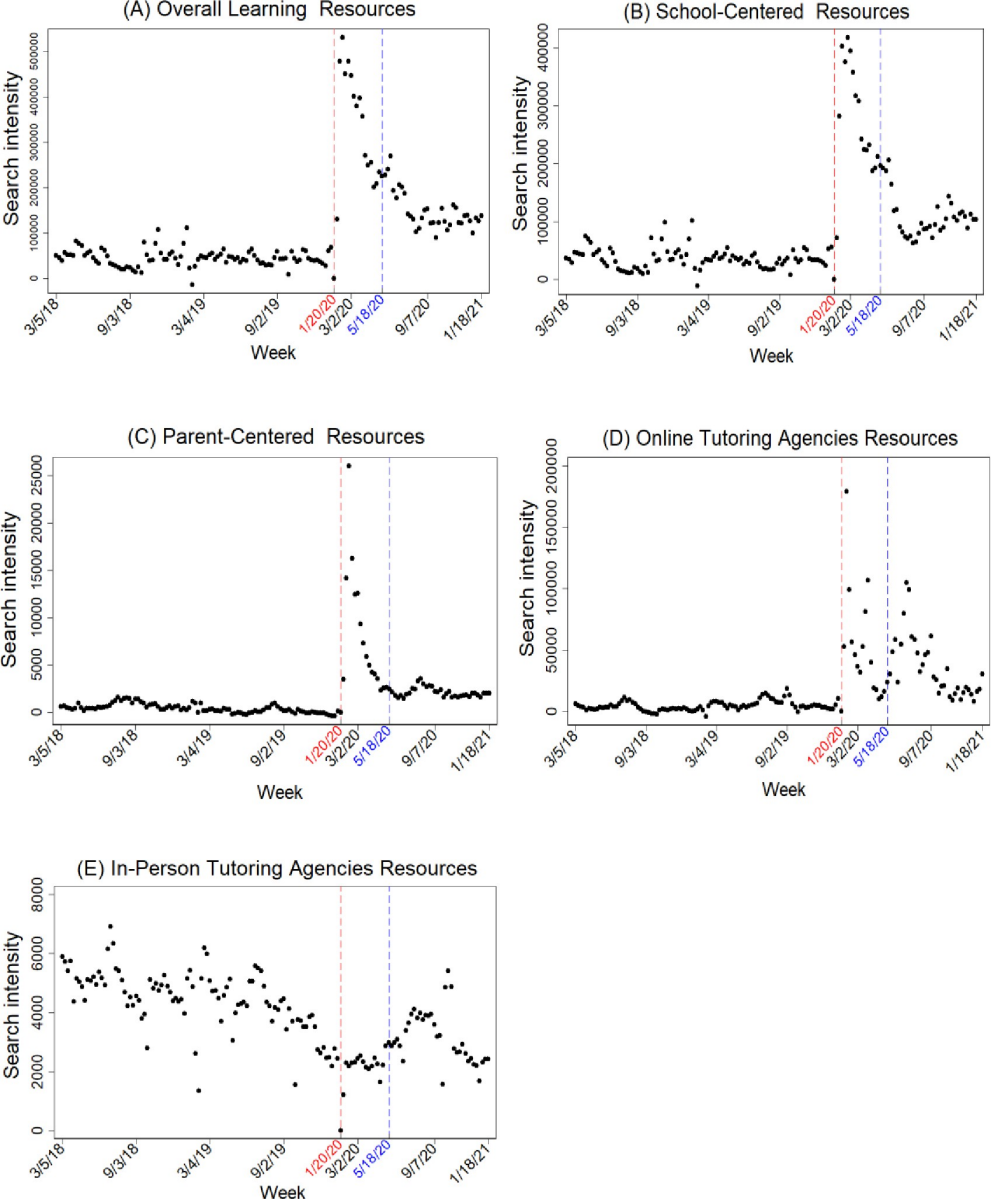
Weekly nationwide search intensity for learning resources. Notes: The figure above shows raw weekly nationwide search intensity relative to intensity on January 20, 2020. The week of January 20, 2020 is the beginning of Wuhan lockdown, and the week of May 18, 2020 is the beginning of school reopening. Panel A shows search intensity for “overall learning resources”. Panel B for “school-centered resources”, panel C for “parent-centered resources”, panel D for “online tutoring agencies resources”, panel E for “in-person tutoring agencies resources”.

In Fig 2 I show the results with nationwide search intensity for learning resources. Panel A shows that the pandemic caused a significant increase in overall search intensity for learning resources. When schools reopened, overall search intensity for learning resources decreased but still doubled compared to the pre-Covid level, suggesting that the pandemic may have a lasting impact on children’s education development.

As indicated in Panels B–D, search intensity for “school-centered resources”, “parent-centered resources”, and “online tutoring agencies resources” peaked in February 2020, more than tripling during school closures compared to the pre-Covid level. This indicates that school lockdowns induced people to use online learning resources to mitigate learning losses. When schools reopened in May, search intensity for these learning resources showed a downward trend but remained higher than the pre-Covid level. By contrast, as shown in Panel E, search intensity for “in-person tutoring agencies resources” decreased during school closures. When schools reopened, search intensity increased slightly but remained lower than the pre-Covid level.

### 5.2 Inequality in access to learning resources

In this subsection, I show the results for how Covid-19 affects the inequality in access to learning resources for households living in high and low SES areas.

#### 5.2.1 Event-study analysis results

**Fig 3:**
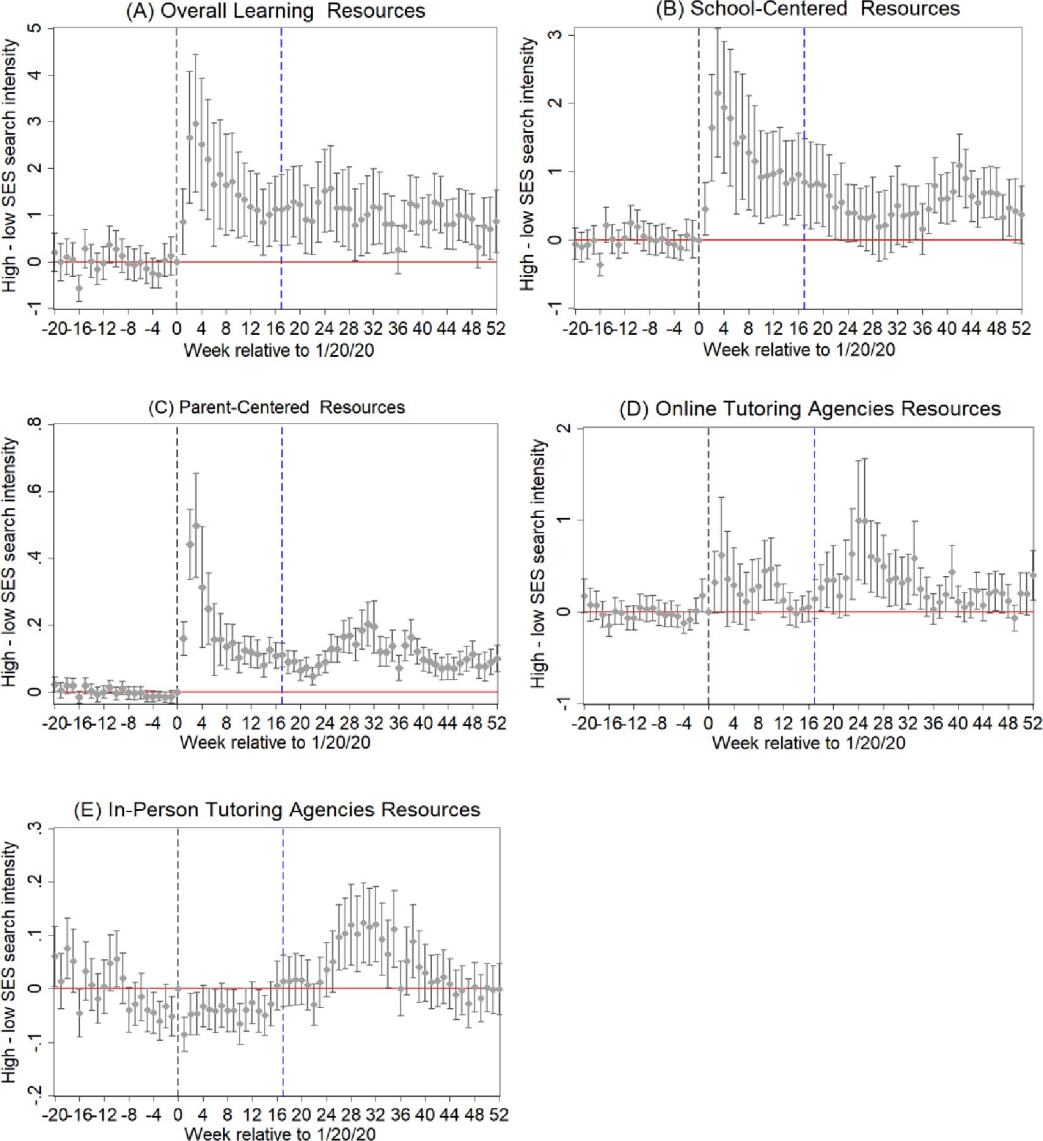
Event study of search intensity gap by socioeconomic status. Notes: This figure shows event study coefficients and corresponding confidence intervals estimating the difference in the weekly value of search intensity between high and low SES areas. The regressions include fixed effects for week of year (1–52) and school year (2011–2021), excluding the week of the Spring Festival. *T* = 17 represents the week when school reopening began. Panel A shows the search intensity for “overall learning resources”, panel B for “school-centered resources”, panel C for “parent-centered resources”, panel D for “online tutoring agencies resources”, panel E for “in-person tutoring agencies resources”.

This study shows the difference in access to learning resources between households living in high and low SES areas in Fig 3. Panel A shows that, in their access to the overall learning resources, High-SES households had an advantage over Low-SES households during school closures. The gap peaked in February 2020, narrowed afterwards but remained significant after schools reopened. This indicates that the pandemic may have long-term effects on educational inequality.

As indicated in Panels B–D, High-SES households had an advantage over Low-SES households in access to school- and parent-centered resources, as well as online tutoring resources during school closures. After schools reopened, these gaps remained significant. One possible explanation is that the shock due to the pandemic permanently changes students’ (parents’, or teachers’) behaviors and thus they still use these online resources even after school reopening [46]. The gap for online tutoring resources increased dramatically during summer vacations following school reopening. In China, many parents perceive supplementary tutoring as an effective way to improve students’ academic performance [38]. This leads to students extensively using tutoring resources during the summer to alleviate the learning loss due to the pandemic, potentially exacerbating the inequality in searching for such resources.

By contrast, Panel E shows that the inequality in access to “in-person tutoring agencies resources” decreased during school closures. This suggests that the suspension of in-person agencies played an important role in alleviating educational inequality during this period. Students from Low-SES households who typically have limited access to such tutoring agencies were temporarily no longer at a disadvantage compared to their peers from High-SES households. But after schools reopened, especially during the summer vacation, this inequality increased.

#### 5.2.2 Difference-in-difference analysis results

I show the difference-in-difference analysis results in Table 2, which were similar to my event study analysis results. In access to the overall learning resources, search intensity increased by 5.005 for Low-SES households but 7.793 for High-SES households during the school closure period, resulting in a 2.788 search gap. After schools reopened, the gap narrowed but remained at 2.152, suggesting that the Covid-19 pandemic may have a lasting impact on inequality in access to learning resources.

**Table 2:** Effects of the pandemic on search intensity by socioeconomic status.

	Overall Learning Resources	School- Centered Resources	Parent- Centered Resources	Online Tutoring Agencies Resources	In-person Tutoring Agencies Resources
	(1)	(2)	(3)	(4)	(5)
SchoolClosure * High SES	7.793***	5.289***	0.723***	1.815***	-0.034***
	(0.323)	(0.233)	(0.019)	(0.093)	(0.007)
SchoolClosure *Low SES	5.005***	3.292***	0.539***	1.157***	0.018***
	(0.286)	(0.228)	(0.016)	(0.069)	(0.006)
SchoolReopen * High SES	4.381***	3.457***	0.227***	0.708***	-0.011
	(0.216)	(0.168)	(0.010)	(0.083)	(0.010)
SchoolReopen * Low SES	2.229***	2.164***	0.122***	-0.018	-0.039***
	(0.189)	(0.157)	(0.007)	(0.051)	(0.009)
High-Low SES Change	2.788***	1.997***	0.184***	0.659***	-0.052***
(SchoolClosure period)	(0.461)	(0.348)	(0.025)	(0.122)	(0.008)
High-Low SES Change	2.152***	1.293***	0.105***	0.726***	0.028*
(SchoolReopen period)	(0.267)	(0.191)	(0.012)	(0.100)	(0.014)
Mean dep.var.	2.203	1.127	0.105	0.763	0.208
N	185,915	185,915	185,915	185,915	185,915

Notes: Each coefficient in the table comes from regressions that interact the SchoolClosure and SchoolReopen indicators with above and below median SES indicators. The “High-Low SES Change” is the estimated difference between high and low SES interaction coefficients. The regressions include fixed effects for week of year (1–52) and school year (2011–2021). The sample contains search data from January 2011 through January 2021. The analysis consists of two periods: The SchoolClosure period (from the end of January to May 2020) and the SchoolReopen period (from June 2020 to January 2021), excluding the week of the Spring Festival. Significance level

*** p*<*0.01

**p*<*0.05

* p*<*0.1.

In access to school-centered resources, parent-centered resources, and online tutoring resources, search intensity increased more for High-SES households than Low-SES households during the school closures period. After schools reopened, the gap in access to school- and parents-centered resources narrowed but remained significant. The gap in access to “online tutoring agencies resources” remained almost unchanged compared to the school closures period level. These results suggest that special attention should be given to students from Low-SES households even after school reopening.

By contrast, in access to “in-person tutoring agencies resources”, search intensity increased for Low-SES households but decreased for High-SES households, resulting in a decreased gap during school closures (-0.052), but this inequality increased after schools reopened.

### 5.3. Robustness

A series of robustness checks show the stability of my results. First, because Hubei province, where the first Covid-19 cases were reported, has been hardest hit by the pandemic, students’ learning behaviors may change dramatically compared with other provinces. I re-estimated my results by excluding Hubei province. As seen in Table 3, results were consistent with my previous DID analysis in Table 2, suggesting that my estimation results were not mainly driven by Hubei province.

**Table 3:** Effects of the pandemic on search intensity, excluding Hubei Province.

	Overall Learning Resources	School- Centered Resources	Parent- Centered Resources	Online Tutoring Agencies Resources	In-person Tutoring Agencies Resources
	(1)	(2)	(3)	(4)	(5)
SchoolClosure * High SES	7.791***	5.321***	0.711***	1.794***	-0.036***
	(0.338)	(0.245)	(0.019)	(0.096)	(0.007)
SchoolClosure *Low SES	4.813***	3.141***	0.530***	1.126***	0.016***
	(0.251)	(0.186)	(0.016)	(0.070)	(0.006)
SchoolReopen * High SES	4.294***	3.393***	0.220***	0.693***	-0.013
	(0.218)	(0.168)	(0.010)	(0.087)	(0.010)
SchoolReopen * Low SES	2.071***	2.048***	0.114***	-0.052	-0.039***
	(0.170)	(0.137)	(0.007)	(0.052)	(0.009)
High-Low SES Change	2.977***	2.181***	0.181***	0.668***	-0.052***
(SchoolClosure period)	(0.452)	(0.330)	(0.025)	(0.126)	(0.008)
High-Low SES Change	2.223***	1.345***	0.106***	0.744***	0.026*
(SchoolReopen period)	(0.276)	(0.197)	(0.012)	(0.104)	(0.014)
Mean dep.var.	2.183	1.124	0.105	0.766	0.210
N	177,160	177,160	177,160	177,160	177,160

Notes: I exclude Hubei Province in our analysis. Each coefficient in the table comes from regressions that interact the SchoolClosure and SchoolReopen indicators with above and below median SES indicators. The “High-Low SES Change” is the estimated difference between high and low SES interaction coefficients. The regressions include fixed effects for week of year (1–52) and school year (2011–2021). The sample contains search data from January 2011 through January 2021. The analysis consists of two periods: The SchoolClosure period (from the end of January to May, 2020) and the SchoolReopen period (from June 2020 to January 2021), excluding the week of the Spring Festival. Significance level

*** p<0.01

**p<0.05

* p<0.1.

Second, I excluded China’s four municipalities (Beijing, Shanghai, Chongqing, and Tianjin) in my analysis to account for the possibility that the increased inequality in access to learning resources may be driven by some big cities with a much higher level of economic and social development. Table 4 shows that the results remained almost unchanged, suggesting that the regional search gap was not caused by individual highly developed cities.

**Table 4:** Effects of the pandemic on search intensity, excluding four municipalities.

	Overall Learning Resources	School- Centered Resources	Parent- Centered Resources	Online Tutoring Agencies Resources	In-person Tutoring Agencies Resources
	(1)	(2)	(3)	(4)	(5)
SchoolClosure * High SES	7.829***	5.299***	0.730***	1.833***	-0.033***
	(0.325)	(0.235)	(0.019)	(0.093)	(0.007)
SchoolClosure *Low SES	5.004***	3.291***	0.538***	1.156***	0.018***
	(0.289)	(0.231)	(0.016)	(0.069)	(0.006)
SchoolReopen * High SES	4.397***	3.464***	0.228***	0.716***	-0.011
	(0.218)	(0.170)	(0.010)	(0.084)	(0.010)
SchoolReopen * Low SES	2.222***	2.165***	0.122***	-0.026	-0.038***
	(0.191)	(0.159)	(0.008)	(0.051)	(0.009)
High-Low SES Change	2.823***	2.008***	0.192***	0.677***	-0.051***
(SchoolClosure period)	(0.464)	(0.351)	(0.025)	(0.123)	(0.008)
High-Low SES Change	2.175***	1.299***	0.106***	0.742***	0.028*
(SchoolReopen period)	(0.269)	(0.193)	(0.012)	(0.100)	(0.015)
Mean dep.var.	2.193	1.127	0.103	0.760	0.204
N	183,855	183,855	183,855	183,855	183,855

Notes: The four municipalities include Beijing, Shanghai, Chongqing and Tianjin. Each coefficient in the table comes from regressions that interact the SchoolClosure and SchoolReopen indicators with above and below median SES indicators. The “High-Low SES Change” is the estimated difference between high and low SES interaction coefficients. The regressions include fixed effects for week of year (1–52) and school year (2011–2021). The sample contains search data from January 2011 through January 2021. The analysis consists of two periods: The SchoolClosure period (from the end of January to May, 2020) and the SchoolReopen period (from June 2020 to January 2021), excluding the week of the Spring Festival. Significance level

*** p<0.01

**p<0.05

* p<0.1.

Third, even though nearly two-thirds of regions close schools in mid-February, people may still be concerned that different school closure time may have an impact on my results. So, I re-estimated my results by limiting the sample to the regions where schools closed on February 10. As shown in Table 5, the results were consistent with my previous DID analysis in Table 2. This suggests that the staggered school closure time concern may be minor.

**Table 5:** Effects of the pandemic on search intensity, limiting the sample to the regions where schools closed on February 10.

	Overall Learning Resources	School- Centered Resources	Parent- Centered Resources	Online Tutoring Agencies Resources	In-person Tutoring Agencies Resources
	(1)	(2)	(3)	(4)	(5)
SchoolClosure * High SES	7.043***	4.708***	0.696***	1.685***	-0.047***
	(0.379)	(0.248)	(0.026)	(0.135)	(0.011)
SchoolClosure *Low SES	5.217***	3.558***	0.495***	1.161***	0.003
	(0.560)	(0.454)	(0.022)	(0.124)	(0.007)
SchoolReopen * High SES	4.174***	3.205***	0.235***	0.724***	0.011
	(0.306)	(0.216)	(0.016)	(0.146)	(0.016)
SchoolReopen * Low SES	2.282***	2.255***	0.109***	-0.027	-0.055***
	(0.364)	(0.214)	(0.012)	(0.069)	(0.014)
High-Low SES Change	1.825**	1.150**	0.201***	0.524***	-0.049***
(SchoolClosure period)	(0.712)	(0.543)	(0.035)	(0.192)	(0.010)
High-Low SES Change	1.893***	0.950***	0.126***	0.751***	0.066***
(SchoolReopen period)	(0.389)	(0.261)	(0.019)	(0.163)	(0.023)
Mean dep.var.	2.140	1.096	0.105	0.728	0.204
N	85,490	85,490	85,490	85,490	85,490

Notes: I limit the sample to the regions where schools closed on February 10 (approximately 46% of the total sample). Each coefficient in the table comes from regressions that interact the SchoolClosure and SchoolReopen indicators with above and below median SES indicators. The “High-Low SES Change” is the estimated difference between high and low SES interaction coefficients. The regressions include fixed effects for week of year (1–52) and school year (2011–2021). The sample contains search data from January 2011 through January 2021. The analysis consists of two periods: The SchoolClosure period (from the end of January to May, 2020) and the SchoolReopen period (from June 2020 to January 2021), excluding the week of the Spring Festival. Significance level

*** p<0.01

**p<0.05

* p<0.1.

Fourth, the school reopening date may vary across China according to local pandemic situations. While schools in most regions reopened by May 18, 2020, schools in several regions reopened later due to small-scale cases of new infections. I excluded regions where schools reopened after May 18 in my analysis, including Beijing, Jilin, Heilongjiang, Hubei, and Ningxia. Table 6 shows that the results remained almost unchanged.

**Table 6:** Effects of the pandemic on search intensity, excluding regions with late reopening.

	Overall Learning Resources	School- Centered Resources	Parent- Centered Resources	Online Tutoring Agencies Resources	In-person Tutoring Agencies Resources
	(1)	(2)	(3)	(4)	(5)
SchoolClosure * High SES	7.802***	5.353***	0.716***	1.773***	-0.040***
	(0.351)	(0.256)	(0.020)	(0.097)	(0.007)
SchoolClosure *Low SES	4.777***	3.106***	0.532***	1.123***	0.017***
	(0.283)	(0.206)	(0.017)	(0.082)	(0.006)
SchoolReopen * High SES	4.270***	3.375***	0.219***	0.690***	-0.014
	(0.233)	(0.179)	(0.011)	(0.094)	(0.010)
SchoolReopen * Low SES	1.959***	1.968***	0.111***	-0.079	-0.040***
	(0.183)	(0.149)	(0.008)	(0.055)	(0.010)
High-Low SES Change	3.025***	2.248***	0.184***	0.650***	-0.057***
(SchoolClosure period)	(0.484)	(0.353)	(0.026)	(0.135)	(0.008)
High-Low SES Change	2.311***	1.407***	0.108***	0.769***	0.027*
(SchoolReopen period)	(0.297)	(0.212)	(0.011)	(0.112)	(0.015)
Mean dep.var.	2.223	1.138	0.106	0.765	0.213
N	162,740	162,740	162,740	162,740	162,740

Notes: I exclude regions where schools reopened after May 18, 2020, including Beijing, Jilin, Heilongjiang, Hubei, and Ningxia. Each coefficient in the table comes from regressions that interact the SchoolClosure and SchoolReopen indicators with above and below median SES indicators. The “High-Low SES Change” is the estimated difference between high and low SES interaction coefficients. The regressions include fixed effects for week of year (1–52) and school year (2011–2021). The sample contains search data from January 2011 through January 2021. The analysis consists of two periods: The SchoolClosure period (from the end of January to May 2020) and the SchoolReopen period (from June 2020 to January 2021), excluding the week of the Spring Festival. Significance level

*** p<0.01

**p<0.05

* p<0.1.

Fifth, people may be concerned that GDP per capita is not a good measure of socioeconomic status (SES) when studying the pandemic impact on educational inequality. Here I used instead the fixed broadband Internet access per capita to measure city-level SES. Table 7 shows that the results were similar to my previous DID analysis in Table 2, where SES was measured by GDP per capita.

**Table 7:** Effects of the pandemic on search intensity by fixed broadband Internet access per capita.

	Overall Learning Resources	School- Centered Resources	Parent- Centered Resources	Online Tutoring Agencies Resources	In-person Tutoring Agencies Resources
	(1)	(2)	(3)	(4)	(5)
SchoolClosure * High SES	7.810***	5.329***	0.712***	1.797***	-0.029***
	(0.330)	(0.253)	(0.020)	(0.086)	(0.007)
SchoolClosure *Low SES	4.788***	3.103***	0.539***	1.130***	0.015***
	(0.258)	(0.187)	(0.014)	(0.073)	(0.006)
SchoolReopen * High SES	4.296***	3.395***	0.223***	0.680***	-0.002
	(0.214)	(0.170)	(0.010)	(0.081)	(0.009)
SchoolReopen * Low SES	2.174***	2.144***	0.119***	-0.039	-0.050***
	(0.179)	(0.148)	(0.008)	(0.045)	(0.009)
High-Low SES Change	3.022***	2.226***	0.173***	0.667***	-0.043***
(SchoolClosure period)	(0.447)	(0.335)	(0.024)	(0.120)	(0.007)
High-Low SES Change	2.122***	1.251***	0.104***	0.719***	0.048***
(SchoolReopen period)	(0.260)	(0.188)	(0.012)	(0.095)	(0.014)
Mean dep.var.	2.203	1.127	0.105	0.763	0.208
N	185,915	185,915	185,915	185,915	185,915

Notes: Each coefficient in the table comes from regressions that interact the SchoolClosure and SchoolReopen indicators with above and below median SES indicators. The “High-Low SES Change” is the estimated difference between high and low SES interaction coefficients. The regressions include fixed effects for week of year (1–52) and school year (2011–2021). The sample contains search data from January 2011 through January 2021. The analysis consists of two periods: The SchoolClosure period (from the end of January to May 2020) and the SchoolReopen period (from June 2020 to January 2021), excluding the week of the Spring Festival. Significance level

*** p<0.01

**p<0.05

* p<0.1.

Finally, people may be concerned that my results are sensitive to the choice of keywords to construct the four types of learning resources. To show the robustness of my results, I reconstructed my measures of these learning resources. Specifically, I excluded keywords in the following situations: (1) keywords also searched by companies and employees for telework during the pandemic (“Zoom” and “DingTalk” in “school-centered resources”); (2) keywords also searched by companies and employees for vocational education or corporate training (“Ambow” in “in-person tutoring agencies resources”); (3) keywords with a large absolute advantage in search intensity (“Xueersi Online School” and “Yuanfudao” in “online tutoring agencies resources”, as seen in Table A5 in S1 File); (4) keywords that only have search data after the pandemic in the Baidu Index (“online course” in “parent-centered resources”). As seen in Table 8, the results were similar to my previous findings in Table 2, suggesting that the keyword choice concern may be minor.

**Table 8:** Effects of the pandemic on search intensity by alternative measures of resources.

	Overall Learning Resources	School- Centered Resources	Parent- Centered Resources	Online Tutoring Agencies Resources	In-person Tutoring Agencies Resources
	(1)	(2)	(3)	(4)	(5)
SchoolClosure * High SES	4.328***	3.202***	0.408***	0.750***	-0.033***
	(0.168)	(0.134)	(0.013)	(0.041)	(0.007)
SchoolClosure *Low SES	2.695***	2.032***	0.309***	0.338***	0.016***
	(0.165)	(0.147)	(0.013)	(0.032)	(0.005)
SchoolReopen * High SES	2.570***	2.337***	0.070***	0.171***	-0.008
	(0.117)	(0.102)	(0.007)	(0.025)	(0.010)
SchoolReopen * Low SES	1.376***	1.549***	0.023***	-0.158	-0.038***
	(0.125)	(0.109)	(0.005)	(0.027)	(0.009)
High-Low SES Change	1.633***	1.170***	0.099***	0.413***	-0.048***
(SchoolClosure period)	(0.247)	(0.207)	(0.018)	(0.055)	(0.007)
High-Low SES Change	1.194***	0.788***	0.047***	0.330***	0.030**
(SchoolReopen period)	(0.132)	(0.103)	(0.011)	(0.034)	(0.014)
Mean dep.var.	1.462	0.665	0.090	0.509	0.198
N	185,915	185,915	185,915	185,915	185,915

Notes: I exclude “Zoom” and “DingTalk” in “school-centered resources”, “online course” in “parent-centered resources”, “Xueersi Online School” and “Yuanfudao” in “online tutoring agencies resources”, and “Ambow” in “in-person tutoring agencies resources”. Each coefficient in the table comes from regressions that interact the SchoolClosure and SchoolReopen indicators with above and below median SES indicators. The “High-Low SES Change” is the estimated difference between high and low SES interaction coefficients. The regressions include fixed effects for week of year (1–52) and school year (2011–2021). The sample contains search data from January 2011 through January 2021. The analysis consists of two periods: The SchoolClosure period (from the end of January to May 2020) and the SchoolReopen period (from June 2020 to January 2021), excluding the week of the Spring Festival. Significance level

*** p<0.01

**p<0.05

* p<0.1.

## 6 Discussion

The literature has well-documented the substantial increase in educational inequality during school closures. However, we know little about whether such an effect persists after school reopening. Several recent studies have indicated that learning loss during school closures did not mechanically disappear as in-person classes resumed [12]. The unequal access to learning resources may be one of the main channels to persistence in widened gaps between students from High- and Low-SES households.

This study explores the persistence of inequality in students’ access to learning resources caused by Covid-19 in China. The results show that the pandemic increased educational inequality among students. High-SES households had better access to school- and parent-centered resources, as well as online tutoring resources, and these gaps persisted after schools reopened. This indicates that disadvantaged students suffer the most from the pandemic, and this inequality may persist over time.

My study has important policy implications. Because school closures may exacerbate educational inequality and such an effect may persist after school reopening, I recommend that countries prioritize policies to compensate for education losses for vulnerable children. Given that countries have almost returned to normal by the end of 2022, governments should pay special attention to disadvantaged students to help them progress better in the post-Covid period.

For instance, given the finding of disparities in access to school-centered resources, the government should invest more in school facilities to equalize learning opportunities. This involvement can take the form of providing resources, infrastructure, and technical support. The findings on tutoring resources highlight the impact of online tutoring agencies resources on increasing educational inequality. This provides insights for developing remedial policies especially in countries that value a tutoring culture. Offering free but high-quality tutoring resources to students living in low-SES areas may effectively enhance their accessibility to such resources. These initiatives have the potential to mitigate educational inequality and contribute to the long-term academic development of students living in Low-SES areas.

It should be noted that this paper used a single source of Baidu index real-time data. While search data has been widely used to predict citizen behavior and decision-making, such as emigration interest [42], environmental protection [47] and changes in well-being [48,49], future research endeavors could combine real-time data with survey data to enhance the robustness of our findings.

## 7 Conclusions

This study explores whether Covid-19 has a lasting impact on educational inequality after school reopening using Baidu Index data from China. I divided learning resources into four categories, covering “school-centered resources”, “parent-centered resources”, “online tutoring agencies resources” and “in-person tutoring agencies resources”. I found that nationwide search intensity for learning resources had more than tripled during school closures compared to the pre-Covid period. When schools reopened, search intensity for learning resources showed a downward trend but remained higher than the pre-Covid level. I also found that the pandemic increased educational inequality among students. High-SES households had better access to school- and parent-centered resources, and online tutoring resources, even after schools reopened. These findings suggest that the Covid-19 pandemic may have a long-term impact on educational inequality.

## Supporting information

S1 File Appendix. (DOCX)

## References

[1] United Nations. Policy brief: Education during COVID-19 and beyond. 2020. [Cited 20 March 2023]. Available from: https://www.un.org/sites/un2.un.org/files/sg_policy_brief_covid-19_and_education_august_2020.pdf.

[2] Azevedo JP, Hasan A, Goldemberg D, Geven K, Iqbal SA. Simulating the potential impacts of COVID-19 school closures on schooling and learning outcomes: A set of global estimates. The World Bank Research Observer. 2021; 36(1):1–40.

[3] Kuhfeld M, Soland J, Tarasawa B, Johnson A, Ruzek E, Liu J. Projecting the potential impact of COVID-19 school closures on academic achievement. Educational Researcher. 2020; 49(8):549–65.

[4] Maldonado JE, De Witte K. The effect of school closures on standardised student test outcomes. British Educational Research Journal. 2022; 48(1):49–94.

[5] Parolin Z, Lee EK. Large socio-economic, geographic and demographic disparities exist in exposure to school closures. Nature human behaviour. 2021; 5(4):522–8. doi: 10.1038/s41562-021-01087-8 33737734 PMC8060162

[6] Horowitz J. Lower-income Parents Most Concerned about their Children Falling behind Amid COVID-19 School Closures. Pew Research Center. 2020.

[7] Bacher-Hicks A, Goodman J, Mulhern C. Inequality in household adaptation to schooling shocks: Covid-induced online learning engagement in real time. Journal of Public Economics. 2021; 193:104345. doi: 10.1016/j.jpubeco.2020.104345 34629567 PMC8486492

[8] Halloran C, Hug CE, Jack R, Oster E. Post COVID-19 Test Score Recovery: Initial Evidence from State Testing Data. National Bureau of Economic Research; 2023 April 10.

[9] Yang J, Huang X, Liu X. An analysis of education inequality in China. International Journal of Educational Development, 2014; 37: 2–10.

[10] Hu H, Lu S, Huang CC. The psychological and behavioral outcomes of migrant and left-behind children in China. Children and Youth Services Review. 2014; 46:1–10.

[11] Singh A, Romero M, Muralidharan K. COVID-19 Learning loss and recovery: Panel data evidence from India. National Bureau of Economic Research; 2022 Oct 17.

[12] Lichand G, Doria CA, Fernandes JPC, Leal-Neto O. Association of COVID-19 incidence and mortality rates with school reopening in Brazil during the COVID-19 pandemic. JAMA Health Forum. 2022; 3: e215032. doi: 10.1001/jamahealthforum.2021.5032 35977276 PMC8903124

[13] Engzell P, Frey A, Verhagen MD. Learning loss due to school closures during the COVID-19 pandemic. Proceedings of the National Academy of Sciences. 2021; 118(17):e2022376118. doi: 10.1073/pnas.2022376118 33827987 PMC8092566

[14] Asanov I, Flores F, McKenzie D, Mensmann M, Schulte M. Remote-learning, time-use, and mental health of Ecuadorian high-school students during the COVID-19 quarantine. World development. 2021; 138:105225. doi: 10.1016/j.worlddev.2020.105225 33110286 PMC7581322

[15] Agostinelli F, Doepke M, Sorrenti G, Zilibotti F. When the great equalizer shuts down: Schools, peers, and parents in pandemic times. Journal of public economics. 2022; 206:104574. doi: 10.1016/j.jpubeco.2021.104574 35017763 PMC8735857

[16] Grewenig E, Lergetporer P, Werner K, Woessmann L, Zierow L. COVID-19 and educational inequality: How school closures affect low-and high-achieving students. European economic review. 2021; 140:103920. doi: 10.1016/j.euroecorev.2021.103920 34602646 PMC8474988

[17] Bray M, Kwok P. Demand for private supplementary tutoring: conceptual considerations, and socio-economic patterns in Hong Kong. Economics of education review. 2003; 22(6):611–20.

[18] Bray M. Benefits and tensions of shadow education: Comparative perspectives on the roles and impact of private supplementary tutoring in the lives of Hong Kong students. Journal of International and Comparative Education. 2013:18–30.

[19] Dawson W. Private tutoring and mass schooling in East Asia: Reflections of inequality in Japan, South Korea, and Cambodia. Asia pacific education review. 2010; 11:14–24.

[20] Kim S, Lee JH. Private tutoring and demand for education in South Korea. Economic development and cultural change. 2010; 58(2):259–96.

[21] Ryu D, Kang C. Do Private Tutoring Expenditures Raise Academic Performance? Evidence from Middle School Students in South Korea. Asian Economic Journal. 2013; 27(1):59–83.

[22] Ager P, Eriksson K, Karger E, Nencka P, Thomasson MA. School closures during the 1918 flu pandemic. Review of Economics and Statistics. 2022:1–28.

[23] Baytiyeh H. Online learning during post-earthquake school closures. Disaster Prevention and Management: An International Journal. 2018; 27: 215–27.

[24] Hansen B. School year length and student performance: Quasi-experimental evidence. Available at SSRN 2269846. 2011 Oct 20.

[25] Liang W, Xue S. Pandemics and Intergenerational Mobility of Education: Evidence from the 2003 Severe Acute Respiratory Syndrome (SARS) Epidemic in China. GLO Discussion Paper Series. 2021.

[26] Meyers K, Thomasson MA. Can pandemics affect educational attainment? Evidence from the polio epidemic of 1916. Cliometrica. 2021; 15(2):231–65. doi: 10.1007/s11698-020-00212-3 32837578 PMC7384283

[27] Bayrakdar S, Guveli A. Inequalities in home learning and schools’ provision of distance teaching during school closure of COVID-19 lockdown in the UK. ISER Working Paper Series; 2020.

[28] Dorn E, Hancock B, Sarakatsannis J, Viruleg E. COVID-19 and student learning in the United States: The hurt could last a lifetime. McKinsey & Company. 2020; 1:1–9.

[29] Jæger MM, Blaabæk EH. Inequality in learning opportunities during Covid-19: Evidence from library takeout. Research in Social Stratification and Mobility. 2020; 68:100524. doi: 10.1016/j.rssm.2020.100524 32834345 PMC7301805

[30] Andrabi T, Daniels B, Das J. Human capital accumulation and disasters: Evidence from the Pakistan earthquake of 2005. Journal of Human Resources. 2021; 0520–10887R1.

[31] Zhang X, Tan Y, Ling Y, Lu G, Liu F, Yi Z, et al. Viral and host factors related to the clinical outcome of COVID-19. Nature. 2020; 583(7816):437–40. doi: 10.1038/s41586-020-2355-0 32434211

[32] Kraemer MU, Yang CH, Gutierrez B, Wu CH, Klein B, Pigott DM, et al. The effect of human mobility and control measures on the COVID-19 epidemic in China. Science. 2020; 368(6490):493–7. doi: 10.1126/science.abb4218 32213647 PMC7146642

[33] Chen J, Chen W, Liu E, Luo J, Song ZM. The economic cost of locking down like china: Evidence from city-to-city truck flows. OpenScholar@ Princeton. 2022:1–43.

[34] Worobey M. Dissecting the early COVID-19 cases in Wuhan. Science. 2021; 374(6572):1202–4. doi: 10.1126/science.abm4454 34793199

[35] Lewis D. Will Omicron finally overpower China’s COVID defences. Nature. 2022; 604(7904):17–8. doi: 10.1038/d41586-022-00884-z 35347306

[36] Zhang X, Zhang W, Chen S. Shanghai’s life-saving efforts against the current omicron wave of the COVID-19 pandemic. The Lancet. 2022; 399(10340):2011–2. doi: 10.1016/S0140-6736(22)00838-8 35533708 PMC9075855

[37] Chen T, Peng L, Yin X, Rong J, Yang J, Cong G. Analysis of user satisfaction with online education platforms in China during the COVID-19 pandemic. Healthcare 2020; 8(3):200. doi: 10.3390/healthcare8030200 32645911 PMC7551570

[38] Zhang Y. Does private tutoring improve students’ National College Entrance Exam performance?—A case study from Jinan, China. Economics of Education Review. 2013; 32:1–28.

[39] UNESCO. Global education monitoring report: non-state actors in education: who chooses? who loses. 2021. [Cited 20 March 2023]. Available from: https://unesdoc.unesco.org/ark:/48223/pf0000379875.

[40] Zhang W, Bray M. Equalising schooling, unequalising private supplementary tutoring: Access and tracking through shadow education in China. Oxford Review of Education. 2018; 44(2):221–38.

[41] Lu J. Investigation on the Present Situation of Extracurricular Training for K12 Students in China. Peking University Open Research Data Platform. 2018.

[42] Qin Y, Zhu H. Run away? Air pollution and emigration interests in China. Journal of Population Economics. 2018; 31(1):235–66.

[43] Kahn ME, Sun W, Zheng S. Clean air as an experience good in urban China. Ecological Economics. 2022; 192:107254. doi: 10.1016/j.ecolecon.2021.107254 34690430 PMC8523121

[44] Huntington-Klein N. The effect: An introduction to research design and causality. CRC Press. 2021.

[45] Corrado CJ. Event studies: A methodology review. Accounting & Finance, 2011; 51(1):207–234.

[46] Barrero JM, Bloom N, Davis SJ. Why working from home will stick. National Bureau of Economic Research; 2021 May 3.

[47] Barwick PJ, Li S, Lin L, Zou E. From fog to smog: The value of pollution information. National Bureau of Economic Research; 2019 Dec 9.

[48] Askitas N, Zimmermann KF. Health and well-being in the great recession. International Journal of Manpower. 2015; 36(1):26–47.

[49] Brodeur A, Clark AE, Fleche S, Powdthavee N. COVID-19, lockdowns and well-being: Evidence from Google Trends. Journal of public economics. 2021; 193:104346. doi: 10.1016/j.jpubeco.2020.104346 33281237 PMC7703221

