# Supplementary material for: Does COVID-19 persistently affect educational inequality after school reopening? evidence from Internet search data in China
Source: PLoS One. 2023 Oct 30;18(10):e0293168. doi: 10.1371/journal.pone.0293168 (PMC10615277; doi:10.1371/journal.pone.0293168)
Supplement: S1 File — (DOCX) [file pone.0293168.s001.docx]

# Supporting information

Table A1. Start and end dates of school lockdowns by province.

| **Province (Including Municipalities)** | **Start Date** | **End Date** |
| --- | --- | --- |
| Beijing | February 17 | August 29 |
| Shanghai | March 2 | May 18 |
| Tianjin | February 10 | May 18 |
| Chongqing | February 10 | April 27 |
| Jiangxi | February 10 | May 18 |
| Shaanxi | February 10 | May 18 |
| Guangxi | February 10 | May 6 |
| Henan | February 10 | May 11 |
| Hebei | February 10 | June 1 |
| Jiangsu | February 10 | May 6 |
| Hunan | February 10 | April 20 |
| Zhejiang | February 10 | April 20 |
| Shandong | February 9 | May 18 |
| Hubei | February 10 | September 1 |
| Guangdong | March 2 | May 11 |
| Heilongjiang | March 2 | June 20 |
| Anhui | March 2 | April 26 |
| Fujian | February 12 | May 11 |
| Shanxi | February 10 | May 18 |
| Sichuan | March 9 | April 13 |
| Liaoning | February 17 | May 18 |
| Jilin | February 24 | June1 |
| Inner Mongolia | March 2 | May 7 |
| Xinjiang | February 17 | March 23 |
| Tibet | March 5 | late March |
| Gansu | February 24 | April 20 |
| Hainan | February 10 | April 20 |
| Qinghai | March 2 | March 25 |
| Yunnan | February 10 | April 26 |
| Guizhou | February 24 | May 15 |
| Ningxia | February 17 | May 27 |

Note：Primary and secondary schools across China reopened at staggered times to ensure students’ safety amid the COVID-19 epidemic. Most secondary schools reopened in early April 2020, and primary schools reopened after May 2020. The “end date” represents the beginning of in-person classes in primary schools. All data come from the official notice from local education bureaus.

Table A2. Four categories of Baidu Index keywords.

| **Category** |  | **Definition** | |  |  | **Keywords in English** |  |  | | **Keywords in Chinese** |  |
| --- | --- | --- | --- | --- | --- | --- | --- | --- | --- | --- | --- |
|  |  |  |  |  |  | Tencent Classroom, Zoom |  |  | | 腾讯课堂,Zoom, | |
|  |  |  |  |  |  | DingTalk, Superstar Learning |  | | | 钉钉,学习通, | |
| **School-Centered** | | online platforms used by schools to | | |  | CCtalk, Zhixue |  |  | | CCtalk,智学网, | |
| **Resources** | | provide instruction | |  |  | Wuxianbao, Leke |  |  | | 无限宝,乐课网, | |
|  |  |  |  |  |  | Xiaoheiban, Classin |  | |  | 晓黑板,Classin | |
|  |  |  |  |  |  | Online Learning, Online Education |  | | | 在线学习,在线教育, | |
|  |  |  |  |  |  | Online Classroom, Network Classroom |  | | | 在线课堂,网络课堂, | |
| **Parent-Centered** | | parents or students are seeking | | |  | Online Courses, Network Courses |  |  | | 在线课程,网络课程, | |
| **Resources** | | supplemental learning resources | | |  | Online Lessons, Online Teaching |  |  | | 网课,网上教学, | |
|  |  |  | | |  | Online Study, Virtual Classroom |  |  | | 网上学习,网上课堂 | |
|  |  |  |  |  |  | Xueersi Online School, Yuanfudao |  | | | 学而思网校,猿辅导, |  |
|  |  |  |  |  |  | Xueersi Peiyou, Koolearn |  |  | | 学而思培优,新东方在线, | |
| **Online Tutoring** | | online tutoring resources | |  |  | 51talk, Genshuixue |  |  | | 51talk,跟谁学, | |
| **Agencies Resources** | | provided by agencies | | |  | Youdao Lessons, 17 Education |  | | | 有道精品课,一起学, | |
|  | |  | | |  | Onion Math, Vipkid |  | | | 洋葱数学,Vipkid, | |
|  |  |  |  |  |  | Jingrui Education , Xueda Education |  | | | 精锐教育,学大教育, |  |
| **In-person Tutoring** | | in-person tutoring resources | |  |  | Only Education, Zhuoyue Education |  | | | 昂立教育,卓越教育, | |
| **Agencies Resources** | | provided by agencies | | |  | Longwen Education, Bangde Education |  |  | | 龙文教育,邦德教育, | |
|  | |  | | |  | Ambow, XSpark Education |  |  | | 安博教育,星火教育, |  |
|  | |  | | |  | Juren Education, Jinghan Education |  |  | | 巨人教育,京翰教育 |  |

Notes: The table above shows the list of all keywords in the four types of learning resources.

Table A3. Weekly search intensity of individual keywords in “school-centered resources”.

| Keywords | Pre-Covid | School Closures |
| --- | --- | --- |
| DingTalk | 22638 | 118671 |
| Superstar Learning | 6289 | 82254 |
| Tecent Classroom | 8170 | 40521 |
| Zoom | 4274 | 26129 |
| Zhixue | 14003 | 11758 |
| Xiaoheiban | 1364 | 7421 |
| Leke | 471 | 6072 |
| Classin | 733 | 4448 |
| Wuxianbao | 150 | 4217 |
| CCTalk | 1274 | 3601 |
| Lejiaolexue | 1219 | 2905 |
| Xueleyun | 709 | 2641 |
| EasiCare | 748 | 1810 |
| Seewo | 1155 | 1465 |
| Khan Academy | 1467 | 1354 |
| Aileqi | 530 | 791 |
| Baijiayun | 261 | 662 |

Notes: Mean weekly nationwide search intensity is shown for pre-Covid and school closure periods. The pre-Covid sample contains search data from February to May 2019. The school closures sample contains search data from February to May 2020.

Table A4. Weekly search intensity of individual keywords in “parent-centered resources”.

| **Keywords** | **Pre-Covid** | **School Closures** |
| --- | --- | --- |
| Online Lessons | 0 | 3417 |
| Online Education | 466 | 1297 |
| Network Classroom | 342 | 1034 |
| Online Classroom | 237 | 948 |
| Online Learning | 444 | 865 |
| Online Study | 178 | 604 |
| Online Teaching | 120 | 534 |
| Network Courses | 246 | 513 |
| Online Courses | 210 | 456 |
| Virtual Classroom | 132 | 349 |
| Online Classroom App | 85 | 207 |
| Online Tutoring | 189 | 199 |
| Educational App | 170 | 154 |
| Practice After Class | 43 | 130 |
| Distance Education Platform | 104 | 123 |
| Online Platforms | 93 | 76 |
| Tutoring Materials | 64 | 18 |
| Distance Learning | / | / |

Notes: Mean weekly nationwide search intensity is shown for pre-Covid and school closure periods. The pre-Covid sample contains search data from February to May 2019. The school closures sample contains search data from February to May 2020. The data of “Distance Learning” is missing in the Baidu index.

Table A5. Weekly search intensity of individual keywords in “online tutoring agencies resources”.

| **Keywords** | **Pre-Covid** | **School Closures** |
| --- | --- | --- |
| Xueersi Online School | 3195 | 23719 |
| Yuanfudao | 2032 | 20600 |
| Genshuixue | 1415 | 3840 |
| Youdao Lessons | 1457 | 3282 |
| 17 Education | 646 | 2869 |
| Onion Math | 1818 | 2740 |
| Xueersi Peiyou | 1199 | 2702 |
| Koolearn | 2618 | 2146 |
| Vipkid | 2950 | 2136 |
| 51Talk | 2703 | 2132 |
| Zhangmen One-To-One | 2270 | 2070 |
| Homework Help Live | 4 | 2029 |
| Tongbuketang | 356 | 1670 |
| Hujiang Online School | 2070 | 1622 |
| 101 Online School | 496 | 1458 |
| Simple Learning Network | 828 | 1143 |
| Acadsoc | 1508 | 1067 |
| Haifeng Education | 1209 | 652 |

Notes: Mean weekly nationwide search intensity is shown for pre-Covid and school closure periods. The pre-Covid sample contains search data from February to May 2019. The school closures sample contains search data from February to May 2020.

Table A6. Weekly search intensity of individual keywords in “in-person tutoring agencies resources”.

| **Keywords** | **Pre-Covid** | **School Closures** |
| --- | --- | --- |
| Xueda Education | 1187 | 913 |
| Only Edycation | 805 | 832 |
| Jingrui Education | 1084 | 675 |
| XSpark Education | 914 | 673 |
| Zhuoyue Education | 835 | 499 |
| Juren Education | 818 | 386 |
| Longwen Education | 477 | 285 |
| Jinghan Education | 445 | 270 |
| Ambow | 277 | 270 |
| Bangde Education | 277 | 217 |
| Siji Education | 297 | 214 |
| Qinxue Education | 227 | 167 |
| U-can Secondary School Education | 154 | 120 |
| Youneng Education | 137 | 73 |

Notes: China has a large number of in-person tutoring agencies, but not many national chains. Mean weekly nationwide search intensity is shown for pre-Covid and school closures periods. The pre-Covid sample contains search data from February to May 2019. The school closures sample contains search data from February to May 2020.


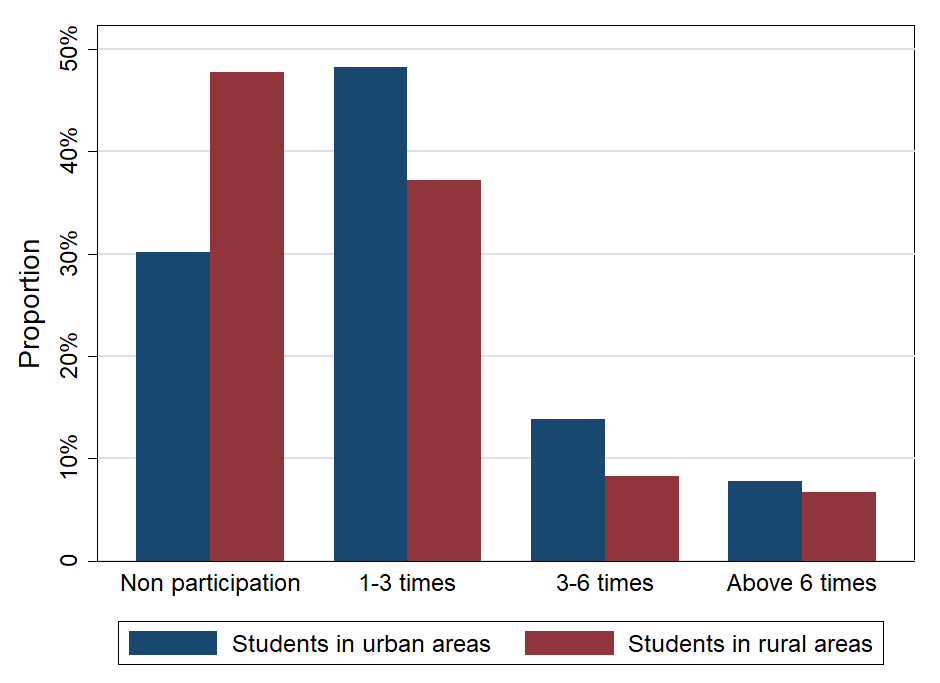


Fig A1. Students’ participation in extracurricular tutoring by region.

Notes: This figure shows the proportion of students’ weekly frequency of participation in extracurricular tutoring in rural and urban areas. I use the survey data from the Present Situation of Extracurricular Training for K12 Students in China.
